# Room-Temperature Structure of Xylitol-Bound Glucose Isomerase by Serial Crystallography: Xylitol Binding in the M1 Site Induces Release of Metal Bound in the M2 Site

**DOI:** 10.3390/ijms22083892

**Published:** 2021-04-09

**Authors:** Ki Hyun Nam

**Affiliations:** Department of Life Science, Pohang University of Science and Technology, Pohang 37673, Korea; structures@postech.ac.kr

**Keywords:** glucose isomerase, xylose isomerase, inhibitor, xylitol, metal binding, metal coordination, serial crystallography, room-temperature structure

## Abstract

Glucose isomerase (GI) is an important enzyme that is widely used in industrial applications, such as in the production of high-fructose corn syrup or bioethanol. Studying inhibitor effects on GI is important to deciphering GI-specific molecular functions, as well as potential industrial applications. Analysis of the existing xylitol-bound GI structure revealed low metal occupancy at the M2 site; however, it remains unknown why this phenomenon occurs. This study reports the room-temperature structures of native and xylitol-bound GI from *Streptomyces rubiginosus* (SruGI) determined by serial millisecond crystallography. The M1 site of native SruGI exhibits distorted octahedral coordination; however, xylitol binding results in the M1 site exhibit geometrically stable octahedral coordination. This change results in the rearrangement of metal-binding residues for the M1 and M2 sites, the latter of which previously displayed distorted metal coordination, resulting in unstable coordination of Mg^2+^ at the M2 site and possibly explaining the inducement of low metal-binding affinity. These results enhance the understanding of the configuration of the xylitol-bound state of SruGI and provide insights into its future industrial application.

## 1. Introduction

Glucose isomerase (GI, D-xylose ketol isomerase; EC 5.3.1.5), also known as xylose isomerase, catalyzes the reversible isomerization of D-glucose and D-xylose to their ketoses D-fructose and D-xylulose, respectively [1]. In GI-catalyzed interconversion, the R-hydroxy aldehyde and R-hydroxy ketone of sugars are isomerized through the formal transfer of two hydrogens [2]. These sugars produced by GI are not only involved in crucial metabolic roles but also widely applied in various industries. In particular, GI is among the most important and highly applicable enzymes employed in the food industry for isomerizing starch-based D-glucose into D-fructose to produce high-fructose corn syrup (HFCS) [3,4,5], which is widely used in food industries, including as a sweetener in soft drinks and other food products, where it replaces beet or cane sugar. Additionally, HFCS is used in the pharmaceutical industry [3,6]. Moreover, the high catalytic activity of GI from *Saccharomyces cerevisiae* has been utilized for the economical production of bioethanol from cellulosic biomass [7,8].

GI has a catalytic domain comprising a (β/α)_8_-barrel, as a TIM-barrel fold, and further forms a tetrameric assembly [9,10,11,12,13]. Bivalent cations, such as Mg^2+^, Co^2+^, or Mn^2+^, are essentially required for the isomerase activity of GI [1,14]. Two metal ions bind to the metal-binding sites M1 and M2 in the active site and act as substrate-binding (M1 site) and catalytic metal (M2 site) ions that promote the hydride-shift mechanism during substrate isomerization [2]. Biochemical studies show that GI activity is reduced by metal ions, such as Ag^+^, Hg^2+^, Cu^2+^, Zn^2+^, and Ni^2+^, as well as by sugar molecules, such as xylitol, arabitol, sorbitol, mannitol, and lyxose [1,15,16]. Among these, xylitol is a pentahydroxy sugar alcohol and a well-known inhibitor of GI activity [9,15,17], which acts as a competitive inhibitor for D-xylose [16].

A previous study solved the crystal structure of xylitol-bound GI from *Streptomyces rubiginosus* (SruGI; PDB code: 5Y4J) [18]. In that study, SruGI crystals containing only one metal at the M1 site were used, and xylitol was stably bound to the M1 site. These results only demonstrated that xylitol is able to bind in one metal-binding mode [18]. During extensive review of xylitol binding to SruGI, evaluation of four other crystal structures of xylitol-bound SruGI (PDB code: 1XIG [19], 2XIS [9], 3GNX (unpublished), and 4DUO [20]) revealed that the metal ion at the M2 site commonly has a higher atom displacement parameter (ADP, also known as B-factor) value than that at the M1 site (Table 1). However, the reason for the low metal occupancy at the M2 site remains unknown. In the present study, two possible scenarios are speculated for low metal occupancy at the M2 site:

(i) Radiation damage of the metal ion at the M2 site by X-ray exposure. In general, data collection using conventional X-ray crystallography produces both global and specific radiation damage [21]. Because elements with a high atomic number (Z) strongly absorb ionizing radiation [22], the electron density of the metal-binding site in SruGI can be affected by X-ray irradiation.
(ii) Xylitol induces low metal occupancy at the M2 site. This indicates that xylitol affects the residues comprising the M2 site and is involved in its binding during the process of maintaining the interaction with the metal of the M1 site.

Of the two hypotheses, radiation damage of metal sites can be minimized using serial crystallographic methods [23,24]. In serial crystallography approaches, multiple crystal samples are continuously delivered to the X-ray interaction position, and the crystal sample is exposed only once to the X-ray [24,25,26], thereby allowing the room-temperature crystal structure to be observed with minimal radiation damage. In particular, this method is advantageous for allowing observation of radiation-sensitive metalloproteins and structural flexibility at room temperature [27,28].

To explain the low metal occupancy at the M2 site of the xylitol-bound state of SruGI, fixed-target serial-millisecond crystallography was performed, and room-temperature structures of native and xylitol-bound SruGI were determined at 1.5 Å and 1.4 Å resolution, respectively. Structural comparison of native and xylitol-bound SruGI showed that xylitol induces a more stable metal-coordination geometry at the M1 site, while distorting the metal coordination geometry of the M2 site. These results not only provide a biologically relevant room-temperature structure of xylitol-bound SruGI with minimized radiation damage through serial crystallography but also offer insights into the molecular mechanism associated with how xylitol affects metal coordination at the M2 site.

## 2. Results

### 2.1. Data Collection

To provide the accurate structural information for the xylitol effect on the M2 site of GI, fixed-target serial-millisecond crystallography was performed at room temperature. For native SruGI, a total of 38,400 images were collected over a period of 1.1 h (Table 2). Among these, 19,461 images contained diffraction patterns, with a crystal hit rate of 50.67%. Of these, 23,552 indexed diffraction patterns were obtained from 15,759 indexed images (Supplementary Figure S1). The indexing rate and multi-crystal hit rate were 80.97% and 49.45%, respectively. Native SruGI data were processed up to 1.5 Å, with an overall completeness, SNR, *R_split_*, and CC of 100, 3.46, 20.10, and 0.9484, respectively.

**Figure 1:**
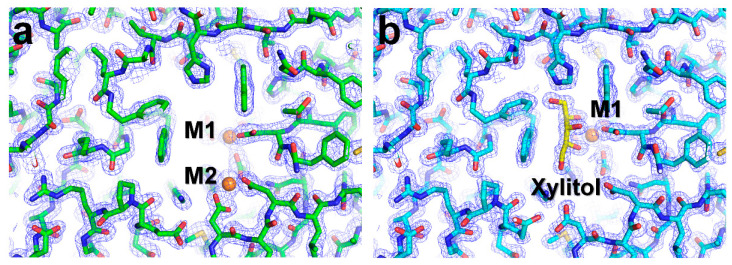
2mFo-DFc electron density map (blue mesh, contoured at 1.3 σ) of the room-temperature structure of (**a**) native and (**b**) xylitol-bound SruGI.

For xylitol-bound SruGI, a total of 59,400 images were collected over a period of 2 h (Table 2). Among these, 11,799 images contained diffraction patterns, with a crystal hit rate of 19.86%. Of these, 8271 indexed diffraction patterns were obtained from 7604 indexed images (Supplementary Figure S2). The indexing rate and multi-crystal hit rate were 64.40% and 8.77%, respectively. Native SruGI data were processed up to 1.4 Å, with an overall completeness, SNR, *R_split_*, and CC of 100, 4.15, 18.33, and 0.9520, respectively. Native and xylitol-bound SruGI crystals displayed similar unit cell parameters and contained one molecule per asymmetric unit in the orthorhombic I222 space group (Table 2). As a consequence, the crystallographic results of native SruGI and xylitol-bound SruGI obtained from soaking in xylitol molecules were almost the same. The electron density maps were obtained by molecular replacement, with both the room-temperature native and xylitol-bound SruGI structures determined at 1.5 Å and 1.4 Å resolution, respectively. The overall electron density of native (Tyr3 to Arg387) and xylitol-bound (Tyr3 to Ala386) SruGI was clearly observed (Figure 1a,b).

### 2.2. Overall Structure

**Figure 2:**
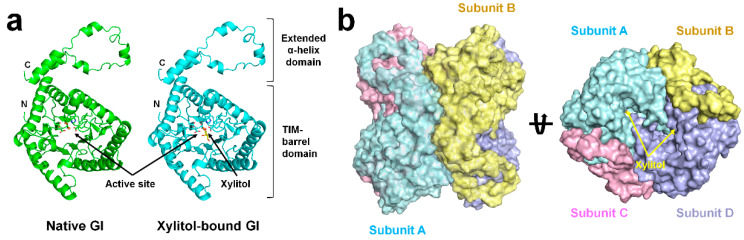
Room-temperature structure of native and xylitol-bound SruGI. Cartoon representation of the (**a**) monomeric and (**b**) tetrameric structures of xylitol-bound SruGI.

The SruGI monomer consisted of 15 α-helices and eight β-strands, which form a TIM-barrel that contains an active site and an extended C-terminal α-helix domain (Figure 2a). The metal-binding site and xylitol molecule are located on the TIM-barrel domain. The monomer subunit further forms the functional tetrameric assembly observed in the crystal symmetry (Figure 2b). The subunit topology and tetrameric assembly of SruGIs were identical with previously reported crystal structures of SruGI [10,11,13,29,30]. The structure of native SruGI was highly similar to other room-temperature crystal structures of native SruGI determined by serial crystallography (PDB codes: 6KCA, 6KD2, 7BVL, and 7CK0) [10,11,29,30], with r.m.s.d. values ranging from 0.088 Å to 0.286 Å (Supplementary Figure S3). Moreover, the structure of xylitol-bound SruGI was similar to other crystal structures of xylitol-bound SruGI determined by traditional X-ray crystallography (PDB codes: 1XIG, 2XIS, 3GNX, 4DUO, and 5Y4J), with r.m.s.d. values ranging from 0.123 Å to 0.273 Å (Supplementary Figure S3). The structures of native and xylitol-bound SruGI in this study were highly similar, with an r.m.s.d. of 0.112 Å and with no significant difference in ADP for the overall structure (Supplementary Figure S3). These results suggested that xylitol-binding caused no significant structural changes to the overall structure of SruGI.

### 2.3. The Metal-Binding Sites and Xylitol-Binding in SruGI

**Figure 3:**
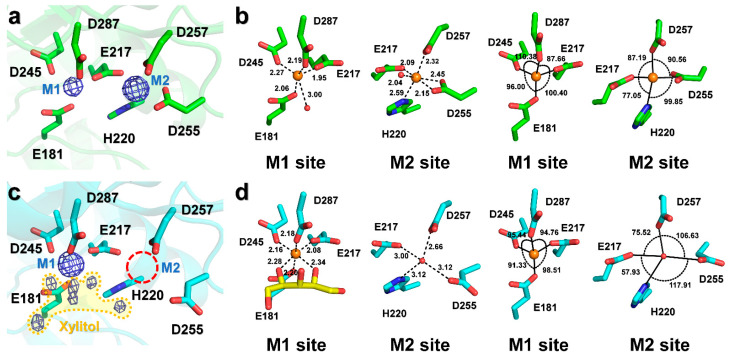
Metal binding sites of native and xylitol-bound SruGI. (**a**) The Fo-Fc omit electron density map (blue mesh, 7σ) for the metal-binding sites of native SruGI. (**b**) Analysis of the interactions and geometries of the M1 (Mg^2+^) and M2 (Mg^2+^) sites of native SruGI. (**c**) The Fo-Fc omit electron density map (blue mesh, 7σ) for the metal-binding sites of xylitol-bound SruGI. The Fo-Fc omit electron density map for xylitol is indicated by yellow area. (**d**) Analysis of the interactions and geometries of the M1 (Mg^2+^) and M2 (water) sites of native SruGI.

Refinement of the structure of room-temperature native SruGI in the absence of metal ions revealed spherical, strong Fo-Fc omit maps (>7 sigma) corresponding to Mg^2+^ ions in both the M1 and M2 sites (Figure 3a and Figure S4), indicating that Mg^2+^ was bound in both the sites. ADP analysis showed that Mg^2+^ ions in the M1 (21.14 Å^2^) and M2 (28.41 Å^2^) sites showed a lower ADP as compared with the overall protein (34.09 Å^2^), indicating that both Mg^2+^ ions were rigidly bound within both the sites. The Mg^2+^ at the M1 site is coordinated by Glu181 (OE2, 2.06 Å), Glu217 (OE2, 1.95 Å), Asp245 (OD2, 2.27 Å), Asp287 (OD2, 2.19 Å), and water molecules (3.00 for W1 and 3.57 Å for W2) (Figure 3b). In the geometry at the M1 site, the bond angles for Glu181 (OE2)-M1-Glu217 (OE2), Glu181 (OE2)-M1-Asp245 (OD2), Glu217 (OE2)-M1-Asp287 (OD2), and Asp245 (OD2)-M1-Asp287 (OD2) were 100.40°, 96.00°, 87.66°, and 110.38°, respectively (Figure 3b). The Mg^2+^ at the M2 site was coordinated by Glu217 (OE1, 2.09 Å), His220 (NE2, 2.59 Å), binate interaction of Asp255 (2.45 Å for OD1 and 2.15 Å for OD2) and Asp257 (OD1, 2.32 Å), and a water molecule (2.04 Å) (Figure 3b). In the geometry at the M2 site, the bond angles of Glu217 (OE1)-M2-His220 (NE2), Glu217 (OE1)-M2-Asp257 (OD1), His220 (NE2)-M2-Asp255 (OD1/OD2), and Asp257 (OD1)-M2-Asp255 (OD1/OD2) were 77.05°, 87.19°, 99.85° (101.06/96.59°), and 90.56° (82.61/98.25°), respectively (Figure 3b).

During structural refinement of xylitol-bound SruGI using native SruGI without metal ions as an initial model, three notable differences in the electron density maps were observed around the metal-binding residues. First, spherical strong Fo-Fc omit maps (>7 sigma) corresponding to the Mg^2+^ ion in the M1 site were observed but none was observed in the M2 site (Figure 3c and Figure S4), which harbored electron density corresponding to a water molecule. The ADP value of the Mg^2+^ (3.82 Å^2^) in the M1 site was lower than that of the entire protein (20.58 Å^2^), whereas that for the water molecule (23.91 Å^2^) in the M2 site was similar to that of the entire protein. These results indicated that Mg^2+^ at the M1 site was strongly bound while displaying low affinity for the M2 site. The Mg^2+^ at the M1 site was coordinated by Glu181 (OE2, 2.20 Å), Glu217 (OE2, 2.08 Å), Asp245 (OD2, 2.16 Å), Asp287 (OD2, 2.18 Å), and two oxygen atoms of xylitol (2.34 Å for O2 and 2.28 Å O4) (Figure 3d). In the M1 site, the bond angles for Glu181 (OE2)-M1-Glu217 (OE2), Glu181 (OE2)-M1-Asp245 (OD2), Glu217 (OE2)-M1-Asp287 (OD2), and Glu245 (OE2)-M1-Glu287 (OE2) were 98.51°, 91.33°, 94.76°, and 95.44°, respectively (Figure 3d). The water at the M2 site was coordinated by Glu217 (OE1, 3.00 Å), His220 (NE2, 3.12 Å), Asp255 (OD2, 3.12 Å), and Asp257 (OD1, 2.66Å) (Figure 3d), which were identical to the metal-binding residues in native SruGI; however, Asp255 demonstrated monodentate coordination of water at the M2 site, whereas Asp255 in native SruGI showed bidentate coordination of Mg^2+^ at this site. In the M2 site, the bond angles for Glu217 (OE1)-W2 (water at the M2 site)-His220 (NE2), Glu217 (OE1)-W2-Asp257 (OD1), His220 (NE2)-W2-Asp255 (OD2), and Asp255 (OD2)-W2-Asp257 (OD1) were 57.93°, 75.52°, 117.91°, and 106.63°, respectively (Figure 3d). Second, negative Fo-Fc electron density was observed around Asp255 that coordinates the metal in the M2 site and positive Fo-Fc electron density was observed in the direction in which the side chain is rotated (Supplementary Figure S5). The electron density map showed partial density for the monodentate coordination by Asp255 in xylitol-bound SruGI as compared with the insufficient density for bidentate coordination. Third, negative Fo-Fc electron density map at the Glu186 side chain was observed and positive Fo-Fc electron density was observed in the direction in which the side chain is rotated (Supplementary Figure S5). These results indicate that xylitol binding to the M1 site induced a conformational change around the M2 site.

**Figure 4:**
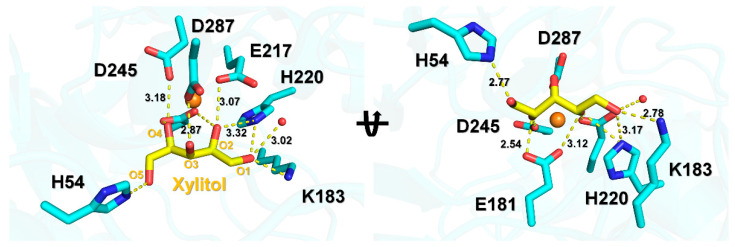
Interaction between xylitol and key residues of SruGI involved in metal binding and catalysis.

In xylitol-bound SruGI, the ADP value of xylitol (14.46 Å^2^) was lower than that for the entire protein (20.58 Å^2^), indicating that the xylitol molecule was strongly bound in the presence of the Mg^2+^ ion in the M1 site. The O1 atom of xylitol interacted with Lys183 (NZ, 2.78 Å) involved in isomerase activity and His220 (NE2, 3.17 Å) and water (3.02 Å) in the M2 site. The O2 atom of xylitol interacts with Glu181 (OE2, 3.12 Å), Glu217 (OE2, 3.07 Å), and Asp287 (OD2, 3.05 Å). The O3 atom of xylitol interacts with Asp287 (OD2, 2.87 Å). The O4 atom of xylitol interacts with Glu181 (OE1, 2.99 Å), Asp245 (OD2, 3.18 Å), and Asp287 (OD2, 2.91 Å). The O5 atom of xylitol interacts with His54 (NE2, 2.77 Å), which plays a role in ring opening of the sugar to the aldose form [2]. These findings indicated that all the oxygen atoms of xylitol molecule tightly interact with the residues involved in metal binding (Glu181, Glu217, His220, Asp245, and Asp287) and enzyme activity (His54 and Lys183) (Figure 4).

### 2.4. Comparison of Native and Xylitol-Bound SruGI

**Figure 5:**
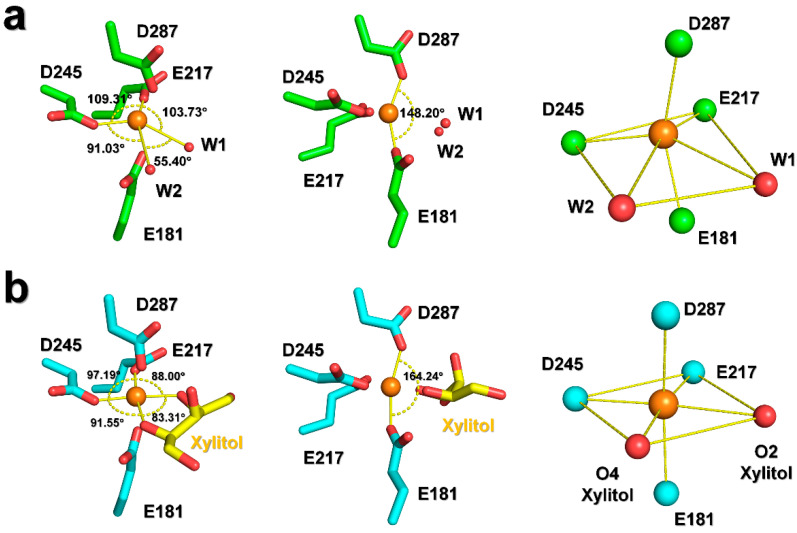
Structural comparison of the geometry of the M1 site in (**a**) native and (**b**) xylitol-bound SruGI.

To identify how the xylitol molecule affects the observed low metal occupancy at the M2 site, the metal-binding sites in xylitol-bound SruGI were compared with those in native SruGI. Because xylitol is bound in the M1 site, this allows it to affect the overall site geometry. Two water molecules were observed around the M1 site in native SruGI, each of which lies at a distance of 3.00 Å and 3.56 Å from the Mg^2+^. These two water molecules with Asp245 (OD2) and Glu217 (OE2) form the octahedral mirror plane, with bond angles for Glu217 (OE2)-M1-Asp245 (OD2), Glu217 (OE2)-M1-W1, Asp245 (OD2)-M1-W2, and W1-M1-W2 of 109.31°, 103.73°, 91.03°, and 55.40°, respectively (Figure 5a). Additionally, the bond angle of Glu181 (OE2)-M1-Asp287 (OD2), which corresponds to the axis of octahedral coordination, is 148.20° (Figure 5a). As a result, the M1 site in native SruGI shows a distorted octahedral coordination. In xylitol-bound SruGI, the water molecules coordinated in the M1 site of native SruGI are replaced by the O2 and O4 atoms of xylitol, which display shorter bond distances (2.34 and 2.28 Å, respectively) from Mg^2+^ in the M1 site. The O2 and O4 atoms of xylitol with Asp245 (OD2) and Glu217 (OE2) form the octahedral mirror plane, with bond angles for Glu217 (OE2)-M1-Asp245 (OD2), Glu217 (OE2)-M1-xylitol (O2), Asp245 (OD2)-M1-xylitol (O4), and xylitol (O2)-M1-xylitol (O4) of 97.19°, 88.00°, 91.55°, and 83.31°, respectively (Figure 5b). The bond angle for Glu181 (OE2)-M1-Asp287 (OD2), which corresponds to the axis of octahedral coordination, is 164.24° (Figure 5b). These findings indicated that when xylitol is bound in the M1 site, this results in a more favorable octahedral coordination of Mg^2+^ (Figure 5).

**Figure 6:**
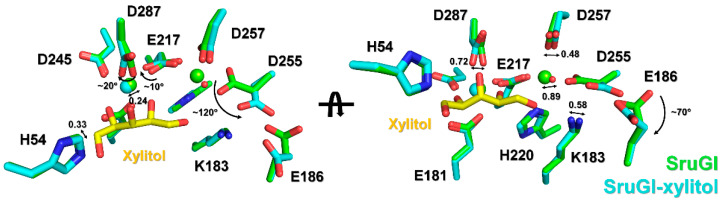
Superimposition of the metal- and xylitol-binding sites of native (green) and xylitol-bound (cyan) SruGI at room temperature.

Accordingly, xylitol binding in the M1 site results in rearrangement of the metal-binding residues for both the M1 and M2 sites in SruGI. Mg^2+^ in the M1 site shifts 0.24 Å towards the center of the M1 octahedron, while the water molecule in the M2 site is shifted 0.89 Å farther from the M1 site, relative to the Mg^2+^ ion in the native structure (Figure 6). Consequently, the centers of both the sites shift away from each other, resulting in separation distances between sites in the native and metal-bound SruGI of 4.96 Å and 5.69 Å, respectively. For the metal-binding residues in the M1 site, there was no significant change in conformation in the side chains of Glu181 and Asp245 responsible for metal-binding platform. By contrast, the Glu217 and Asp287 side chains were rotated by ~10° and 20°, respectively, toward the metal in the M1 site, which resulted in a shift <0.77 Å in the atoms responsible for metal-binding (Figure 6). In the M2 site, the side chain of Glu217 involved in metal coordination was shifted 0.51 Å toward the M1 site. Moreover, the Asp257 side chain shifted 0.48 Å toward the M2 site, whereas that of His220 shifted 0.40 Å in the direction of the bound xylitol in the M1 site. Specifically, Asp255 side chain was rotated ~120°, which shifted the orientation of the metal-binding atom >2.0 Å relative to the position observed in native SruGI. Furthermore, the Glu186 side chain was rotated by ~67° away from the M2 site, which appeared to be necessary to avoid steric hindrance with the altered orientation of the Asp255 side chain. This result demonstrated that xylitol binding with the metal bound in the M1 site has a structural effect on not only the M1 site but also the M2 site and its surrounding residues.

## 3. Discussion

GI is an important enzyme commonly used in the production of HFCS and bioethanol. Biological and structural analyses of GI substrates or inhibitors, such as xylitol, provide important information that can be employed in enzyme engineering for industrial applications. This study was conducted to determine why metal occupancy in the M2 site is low in xylitol-bound SruGIs. The hypotheses were that this was an artefact of X-ray-related radiation damage to the metal bound in the M2 site or structural changes to the M2 site conformation by xylitol. To test the first hypothesis, room-temperature structures for both native and xylitol-bound SruGI were determined by serial crystallography in order to minimize radiation damage. The native SruGI structure clearly showed electron density corresponding to the Mg^2+^ in both M1 and M2 sites. This suggested that radiation did not affect metal occupancy in the M2 site. For the second hypothesis, comparison of the native structure with the xylitol-bound structure showed variations in bond length and angles between the metal and metal binding residues at the M1 site. Specifically, the bond lengths and angles associated with the octahedral geometry ranged from 2.00 Å to 2.20 Å and from 81.47° to 106°, respectively, whereas those for the M2 site ranged from 2.10 Å to 2.60 Å and from 76.91° to 99.96°, respectively. This indicated that the M1 site demonstrated a higher-affinity interaction with Mg^2+^ than the M2 site, whereas in terms of octahedral geometry, the bond angles in the M2 site appeared more stable. Upon xylitol binding to the M1 site, configuration changes in the metal-binding residues of both the sites were observed. In particular, the Mg^2+^ in the M1 site shifted 0.28 Å toward the Asp245 side chain, and the Glu217 side chain shifted 0.30 Å toward the bound Mg^2+^ and farther from the Mg^2+^ bound in the M2 site by 0.60 Å relative to the native SruGI structure. This resulted in a Glu217-Mg^2+^-Asp245 bond angle that was close to an ideal octahedral geometry (105.91° to 98.65°). Thus, the xylitol-bound SruGI shows bond lengths and angles between the metal-binding residues and Mg^2+^ in the M1 site of 1.97 Å to 2.20 Å and 92.81° to 98.65°, respectively, versus 2.57 Å to 3.36 Å and 101.22° to 112.12° in M2, respectively. As a result, when xylitol binds to the M1 site, metal binding remains strong relative to that observed in native SruGI, whereas the water molecules bound in the M2 site demonstrate weak affinity and distorted octahedral geometry.

**Figure 7:**
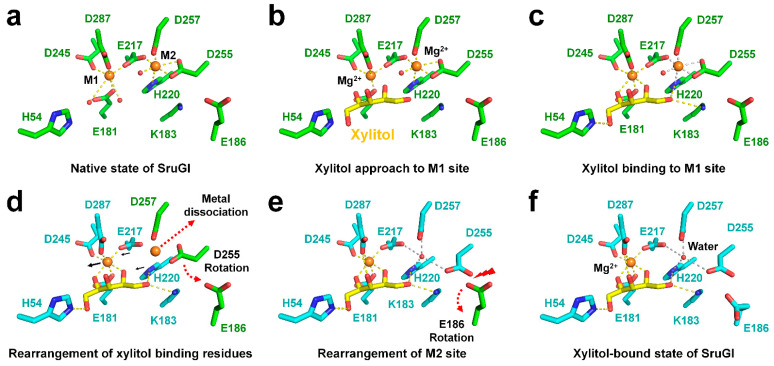
Proposed mechanism describing the effect of xylitol binding in the M1 site on the M2 site in SruGI. (**a**) The two metal-binding modes of native SruGI. (**b**) Xylitol binding to the M1 site of SruGI, where it (**c**) interacts with Mg^2+^. (**d**) Rearrangement of the M1 site and xylitol-binding residues (cyan), resulting in the formation of stable octahedral coordination of Mg^2+^ while the geometry of the M2 site is distorted. (**e**) Mg^2+^ is released from M2 by rearrangement of metal-binding residues around the M2 site, including altered orientation of the Asp255 side chain toward Glu186. Conformational changes in the Glu186 side chain occur in response to steric hindrance with Asp255. (**f**) Xylitol-bound state of SruGI.

The room-temperature structures of native and xylitol-bound SruGI offered insights into the mechanism underlying metal release at the M2 site by xylitol binding to the M1 site (Figure 7). In native SruGI, the Mg^2+^ bound in the M1 and M2 sites maintains octahedral geometry through interactions with Glu217. Upon xylitol binding to the Mg^2+^ in the M1 site, rearrangement of xylitol-binding residues does not alter this geometry in M1, whereas coordination of the Mg^2+^ in M2 is changed through interactions with Glu217 and His220 located proximal to the M1 site. This resulted in distortion of the M2 site, thereby causing unstable coordination of Mg^2+^, subsequent release of Mg^2+^ from M2, and a conformational change in Asp255 and Glu186.

**Figure 8:**
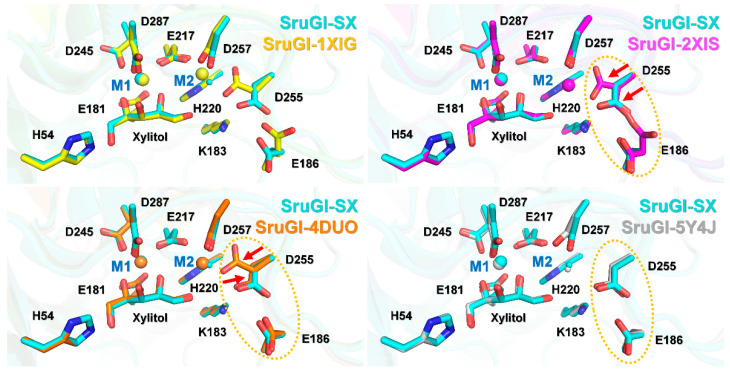
Comparison of the structures of room-temperature xylitol-bound SruGI (SruGI-SX, cyan) determined by serial crystallography and xylitol-bound SruGI variants (PDB code: 1XIG (yellow), 2XIS (pink), 4DUO (orange), and 5Y4J (gray)) determined by traditional X-ray crystallography. In 2XIS and 4DUO, two alternate conformations of D255 are indicated by red arrows.

To date, five xylitol-bound SruGI structures (PDB codes: 1XIG, 2XIS, 3GNX, 4DUO, and 5Y4J) have been determined using traditional X-ray crystallography. These structures provide useful information for understanding the xylitol-bound state of SruGI; however, they are less biologically relevant because of radiation damage associated with use of the X-ray. Moreover, in the 3GNX structure, the xylitol and metal ions were not accurately oriented according to the electron density map [18]. Therefore, the room-temperature xylitol-bound SruGI determined by serial crystallography potentially provides more biologically relevant structural information. For comparison, the structure of xylitol-bound SruGI determined by serial crystallography was superimposed with the structures of xylitol-bound variants previously determined using traditional crystallographic techniques (PDB codes: 1XIG, 2XIS, 4DUO, and 5Y4J) (Supplementary Figure S6). The results revealed clear differences in the conformations of the M1 and M2 sites (Figure 8). However, given the differences in crystallization conditions and data-collection environments, direct structural comparisons are inappropriate. For example, the metal ions in sites M1 and M2 in the 1XIG and 3GNX structures were modeled with Mn^2+^ rather than with Mg^2+^, used in the present study. Additionally, the final structures of 1XIG, 2XIS, 3GNX, and 4DUO were refined with a metal ion bound in the M2 site, which affects the conformation of neighboring residues.

Nevertheless, in the 2XIS, 4DUO, and 5Y4J structures, the Glu186 and Asp255 side chains changed conformations upon xylitol binding to M1 (Figure 8). As noted, the ADP values of the metals in the M2 site of the xylitol-bound 1XIG, 2XIS, 3GNX, and 4DUO structures were higher than those for the overall proteins (Table 1), suggesting that the metal bound in the M2 site in xylitol-bound SruGI is released or has low metal occupancy.

Accordingly, re-refinement of the 3GNX and 4DOU structures was performed, where the bound metals were replaced with water molecules in the M2 site using the deposited structure factors from PDB (structure factors of 1XIS and 2XIS are not available in PDB). The results showed ADP values of water at M2 site of 3GNX and 4DOU close to those of the overall protein along with the absence of positive Fo-Fc electron density map corresponding to the metals (Table 3 and Supplementary Figure S7). These findings indicate that the metal bound in the M2 site of previously determined xylitol-bound SruGIs might not be fully occupied or water binding, which is consistent with our findings related to the xylitol binding inducing the release of the metal bound in the M2 site.

On the contrary, the high ADP value of the M2 site of SruGI may have an effect because of incorrect assessment of the metal cation. To confirm this, Mg^2+^ or Mn^2+^ was added to the metal sites of SruGI-SX, 3GNX, 4DUO, and 5YJA, followed by refinement, and evaluated using the CheckMyMetal server. In all the xylitol-bound SruGI structures, it was confirmed that the M2 site still showed a high ADP value (Supplementary Table S1). This indicates that the metal is not fully occupying the M2 site of xylitol-bound SruGI.

These observations of the possible mechanism of metal release from the M2 site by xylitol binding to the M1 site of GI at room temperature is useful for industrial applications. For example, binding of xylitol or a similar inhibitor to M1 will release the metal bound in the M2 site, resulting in no isomerization activity of GI even if the inhibitor is subsequently released from the M1 site. Use of this configuration in the food or bioethanol industry will require removal of the inhibitor and subsequent addition of metal ion to allow binding to the M2 site in order to promote catalysis.

## 4. Materials and Methods

### 4.1. Sample Preparation

Glucose isomerase from *Streptomyces rubiginosus* (Cat. No. HR7-102) was purchased from Hampton Research (Aliso Viejo, CA, USA) as a crystal suspension containing crystals of various sizes (5–60 μm) in a solution of 6 mM Tris-HCl, pH, 7.0, 0.91 M (NH_4_)_2_SO_4_, and 1 mM MgSO_4_. This crystal suspension was used directly for X-ray data collection without further purification. The crystal suspension (200 μL) was transferred to a 1.5 mL microcentrifuge tube using a pipette and left at room temperature for 20 min to allow the crystals to settle. The supernatant was then removed so that the volume ratio of crystals and supernatant was 1:1. A nylon mesh-based sample holder was used for sample delivery during fixed-target serial crystallography as previously reported [31]. The 30 μL crystal suspension was loaded into a nylon-mesh-based sample holder (dimension: 8 × 8 mm) with a nylon mesh of 60 μm pore size. After spreading the crystal sample evenly over the film with a pipette tip, 10 μL of the supernatant was removed using a pipette. Subsequently, a second polyimide film was used to cover the sample holder to prevent dehydration of the crystal sample. For preparation of xylitol-bound SruGI crystals, the crystal supernatant was transferred into a 1.5 mL microcentrifuge tube, centrifuged at 12,032× *g* for 5 min, and the supernatant was transferred to another microcentrifuge tube. The xylitol powder (Cat No. X3375; Sigma-Aldrich, St. Louis, MO, USA) was dissolved in the supernatant to reach a final concentration of xylitol solution at 20 mM. This xylitol solution was added to the crystal suspension and incubated for 1 h. The method used to load the sample onto the nylon mesh-based sample holder was the same as that for native SruGI sample preparation.

### 4.2. Data Collection

Fixed-target serial millisecond crystallography experiments were performed at beamline 11C at the Pohang Accelerator Laboratory (Pohang, South Korea) [32]. The beam size of the focused X-ray at the sample position was 6.5 µm (vertical) × 8.5 µm (horizontal) full width at half maximum. The X-ray wavelength and flux were 0.9795 Å and 1.2 × 10^12^ photons/sec, respectively. The nylon mesh-based sample holder containing the crystal samples was mounted on the goniometer. Raster scans were performed at a 50 μm scan interval in both vertical and horizontal directions. The data collection strategy was similar to that reported previously [33]. The crystals were exposed to the X-ray beam for 100 ms, with an oscillation of 0.007° at each raster scan point. Diffraction data were recorded with a PILATUS 6M detector (Dectris, Baden-Dättwil, Switzerland) with a 10 Hz readout. Diffraction datasets were collected at room temperature (25 °C). Data collection statistics are shown in Table 2.

### 4.3. Data Processing and Structure Refinement

Among the total collected images, hit images containing >20 Bragg’s peaks with signal-to-noise ratios (SNRs) >5 were filtered using the Cheetah program [34]. These images were further indexed and scaled using the CrystFEL program [35]. The phasing problem was solved using the molecular-replacement method, as implemented in MOLREP [36], and the crystal structure of SruGI (PDB code: 7CK0) [29] as the search model. Model building was performed using the COOT program [37], and structural refinement was performed using REFMAC5 [38]. The geometries of the final models were checked using MolProbity [39]. Refinement statistics are shown in Table 2. The figures were generated by PyMOL (Schrödinger, LLC, New York, NY, USA). The structure factor and coordinate files were deposited in the Protein Data Bank under the accession code 7DFJ (native SruGI) and 7DFK (xylitol-bound SruGI)]. The diffraction images were deposited in CXIDB under ID 173 (native SruGI) and 174 (xylitol-bound SruGI).

### 4.4. Re-Refinenment of the Deposited Xylitol-Bound SruGI Structure

The structure factors and coordinates for xylitol-bound SruGI (PDB code: 3GNX and 4DUO) were download from the Protein Data Bank. After replacing the metal at the M2 site with water molecules, re-refinement was performed through REFMAC5 [38]. The metal binding sites were validated using the CheckMyMetal (CMM) server [40].

## 5. Conclusions

In summary, the room-temperature structures of native and xylitol-bound SruGI were determined using serial millisecond crystallography in order to avoid possible radiation damage and to obtain a more biologically relevant structure than currently available. Structural comparison of native and xylitol-bound SruGI suggested that xylitol binding in the M1 site induces the release of the metal bound in the M2 site and involved in enzyme activity. These results provide insights into the effect of the xylitol inhibitor on the SruGI activity and its possible future industrial applications.

## Figures and Tables

**Table 1:** Atomic displacement parameter (ADP) statistics for the xylitol-bound SruGIs by traditional X-ray crystallography.

PDB Code	Temperature (K)	B-Factor (Å^2^)				Reference
		Protein	Xylitol	M1 Site	M2 Site	
1XIG	285–287 or 277–279	9.96	5.35	6.92 (Mn^2+^)	21.11 (Mn^2+^)	[19]
2XIS	289.15–293.15	13.40	12.51	10.41 (Mg^2+^)	41.55 (Mg^2+^)	[9]
3GNX ^a^	293	14.26	14.61	12.73 (Mn^2+^)	49.62 (Mn^2+^)	unpublished
		14.51	18.34	12.00 (Mn^2+^)	41.10 (Mn^2+^)	
4DUO	293	22.07	14.60	5.46 (Mg^2+^)	42.81 (Mg^2+^)	[20]
5Y4J	100	11.87	7.13	6.02 (Mg^2+^)	9.71 (Water)	[18]

^a^ Two SruGI molecules are occupied in asymmetric unit.

**Table 2:** Data collection and refinement statistics for native and xylitol-bound SruGI.

Data Collection	SruGI	SruGI-Xylitol
Wavelength (Å)	0.9795	0.9795
Temperature (°C)	25	25
Detector	Pilatus 6M	Pilatus 6M
Collected images	38,400	59,400
Hit images	19,461	11,799
Indexed images	15,759	7604
Indexed pattern	23,552	8271
Space group	I222	I222
a, b, c (Å)	93.94, 99.60, 102.92	94.19, 99.92, 103.25
α, β, γ (°)	90, 90, 90	90, 90, 90
Resolution range (Å)	71.9–1.50 (1.55–1.50)	72.4–1.40 (1.45–1.40)
No. of unique reflections	77,277 (7649)	95,564 (9462)
Completeness (%)	100.0 (100.0)	100.0 (100.0)
Redundancy	736.2 (281.2)	277.9 (112.7)
SNR	3.46 (1.13)	4.15 (1.10)
CC	0.9484 (0.7026)	0.9520 (0.5968)
CC*	0.9866 (0.9084)	0.9876 (0.8646)
R_split_^a^	20.10 (86.43)	18.33 (98.38)
Wilson B factor (Å^2^)	27.0	15.0
Refinement		
Resolution range (Å)	71.57–1.50	71.78–1.40
R_work_^b^	0.2233	0.2350
R_free_^c^	0.2480	0.2505
R.m.s. deviations		
Bonds (Å)	0.009	0.015
Angles (°)	1.024	1.279
Average B factors (Å^2^)		
Protein	34.09	20.58
M1 site M2 site	28.41 (Mg^2+^) 21.14 (Mg^2+^)	3.82 (Mg^2+^) 23.91 (water)
Xylitol		14.46
Water	41.40	29.93
Ramachandran plot		
Most favored (%)	97.12	97.12
Allowed (%)	2.62	2.62
Outliers (%)	0.26	0.26

Values for the outer shell are given in parentheses. ^a^ *R_split_* = (12) ∑hkl|Ihkleven−Ihklodd|12|Ihkleven−Ihklodd|; ^b^ *R_work_* = Σ||F_obs_|-|F_calc_||/Σ|F_obs_|, where F_obs_ and F_calc_ are the observed and calculated structure-factor amplitudes, respectively. ^c^ *R_free_* was calculated as *R_work_* using a randomly selected subset (10%) of unique reflections not used for structure refinement.

**Table 3:** Atomic displacement parameter (ADP) of re-refined xylitol-bound SruGI by traditional X-ray crystallography.

PDB Code	B-Factor (Å^2^)					Reference
	Protein	Xylitol	M1 Site	M2 Site	Water	
SruGI-SX	20.58	14.46	3.82 (Mg^2+^)	23.91 (Water)	29.93	This study
Deposited 3GNX ^a^	14.26	14.61	12.73 (Mn^2+^)	49.62 (Mn^2+^)	23.24	Unpublished
	14.51	14.51	12.00 (Mn^2+^)	41.10 (Mn^2+^)	22.72	
Re-refined 3GNX	16.84	11.13	13.16 (Mn^2+^)	16.24 (Water)	25.07	This study
	17.08	12.54	12.91 (Mn^2+^)	27.70 (Water)	24.94	
Deposited 4DUO	22.07	14.46	5.46 (Mg^2+^)	42.81 (Mg^2+^)	34.13	[20]
Re-refined 4DUO	17.92	10.22	5.96 (Mg^2+^)	28.40 (Water)	25.82	This study

^a^ Two SruGI molecules are occupied in asymmetric unit.

## Data Availability

The data that support the findings of this study are available from the corresponding author upon reasonable request.

## Supplementary Materials

The following are available online at https://www.mdpi.com/article/10.3390/ijms22083892/s1, Figure S1: Diffraction image of native SruGI. Figure S2: Diffraction image of xylitol-bound SruGI. Figure S3: Structural comparison of crystal structures of SruGIs. Figure S4: The Fo-Fc omit electron density map for the metal-binding site of native and xylitol-bound SruGI. Figure S5: 2Fo-Fc and Fo-Fc electron density map of xylitol-bound SruGI during the structure refinement. Figure S6: Superimposition of xylitol-bound SruGI. Figure S7: Re-refinement of the deposited xylitol-bound SruGIs. Table S1. Atomic displacement parameter (ADP) of the re-refined of metal-binding site of xylitol-bound SruGIs.
