# Supplementary material for: Room-Temperature Structure of Xylitol-Bound Glucose Isomerase by Serial Crystallography: Xylitol Binding in the M1 Site Induces Release of Metal Bound in the M2 Site
Source: Int J Mol Sci. 2021 Apr 9;22(8):3892. doi: 10.3390/ijms22083892 (PMC8070043; doi:10.3390/ijms22083892)
Supplement: Supplementary file 1 [file ijms-22-03892-s001.pdf]

Supplementary Data

Room Temperature Structure of Xylitol-bound Glucose Isomerase by Serial Crystallography: Xylitol Binding in the M1 Site Induces Release of Metal Bound in the M2 Site

Ki Hyun Nam*

Department of Life Science, Pohang University of Science and Technology, Pohang, 37673, Korea

*Correspondence: structures@postech.ac.kr

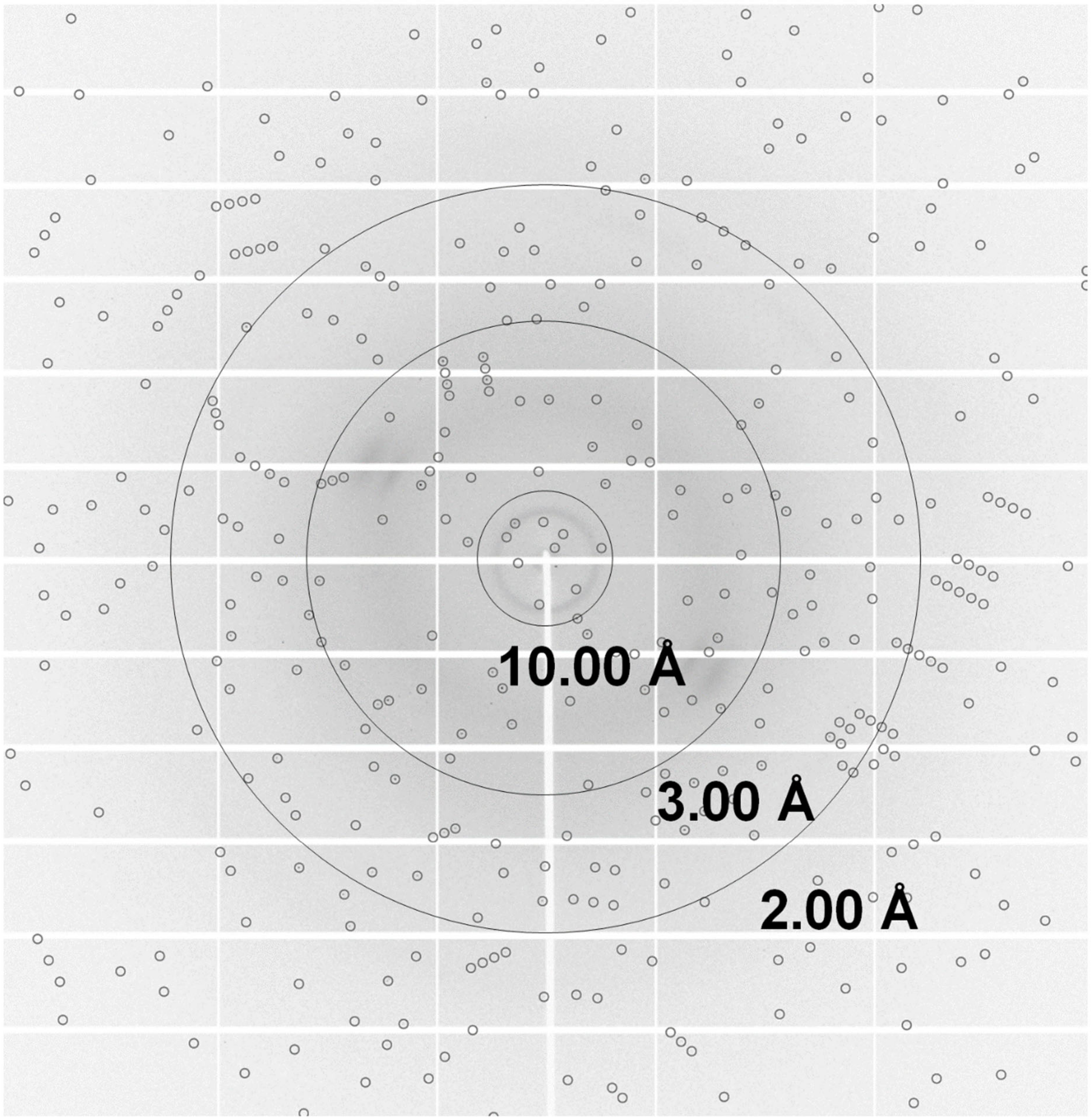

Supplementary Figure S1. Typical diffraction image of native SruGI according to serial-millisecond crystallography. The circles indicate the predicted peak positions.

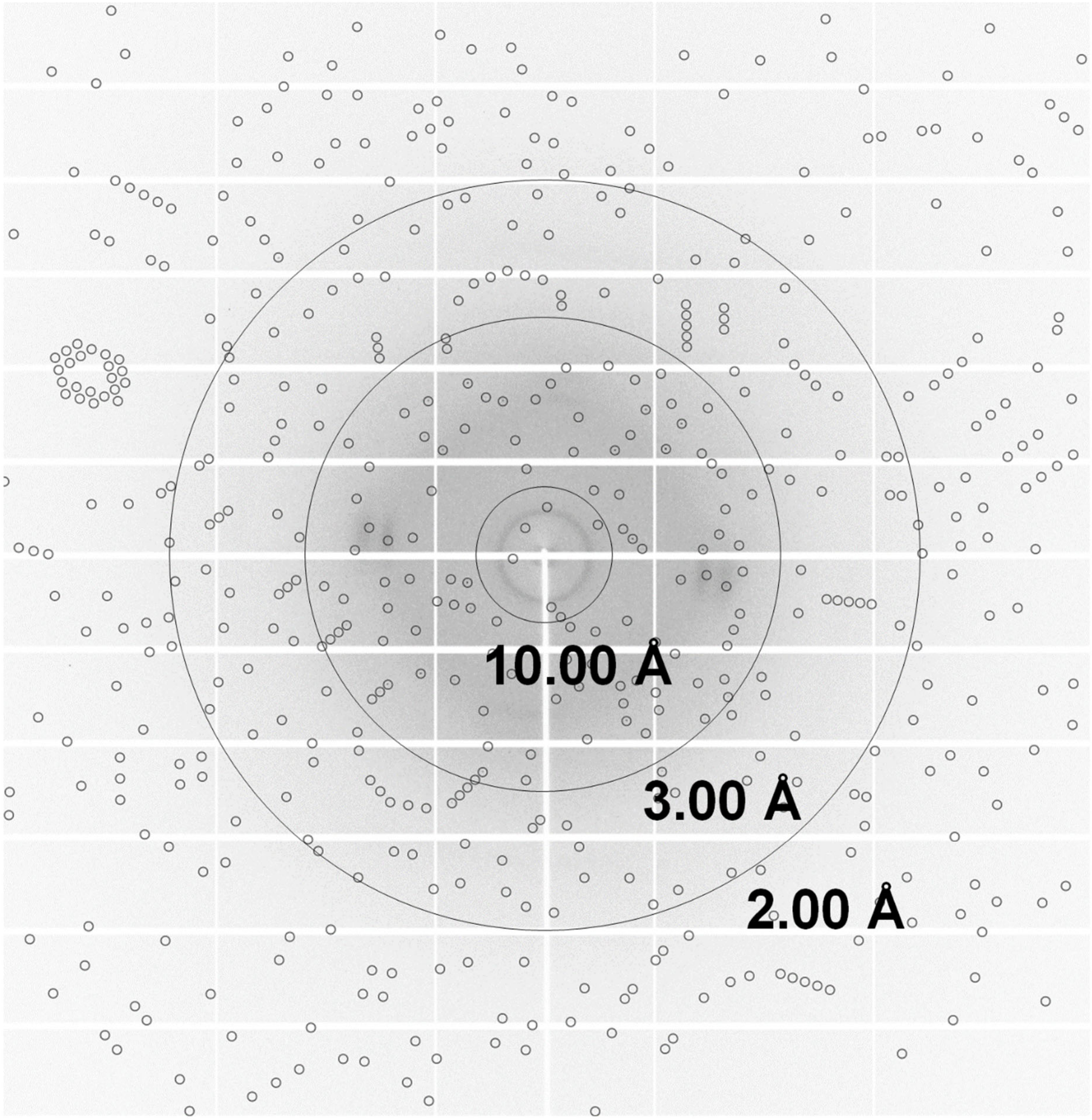

Supplementary Figure S2. Typical diffraction image of xylitol-bound SruGI according to serial-millisecond crystallography. The circles indicate the predicted peak positions.

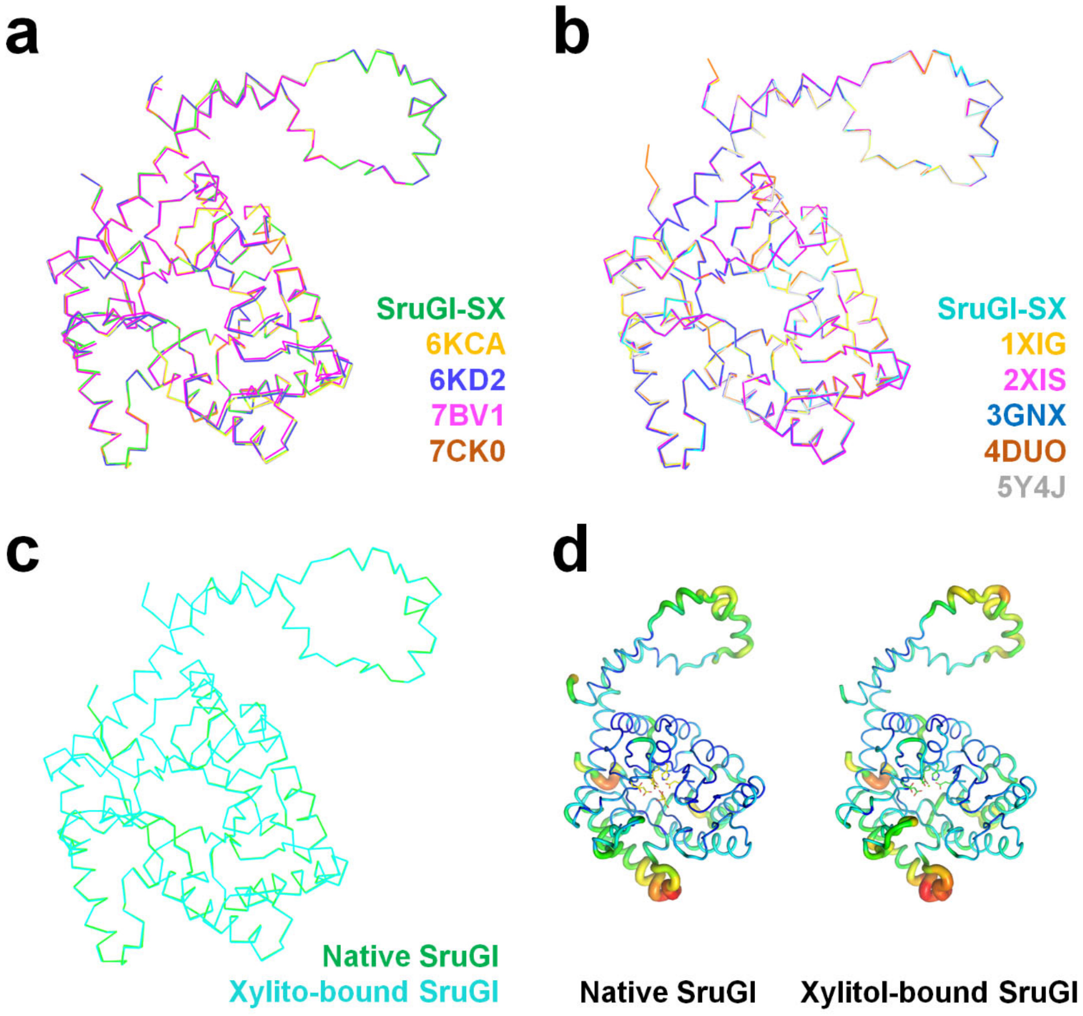

Supplementary Figure S3. Structural comparison of crystal structures of SruGIs. (a) Superimposition of native SruGI determined by serial millisecond crystallography with other room temperature structure of native SruGI (PDB code: 6KCA, 7BV1, 6KD2 and 7CK0). (b) Superimposition of xylitol-bound SruGI determined by serial millisecond crystallography with other crystal structure of xylitol-bound SruGI (1XIG, 2XIS, 3GNX, 4DUO and 5Y4J). (c) Superimposition of native (green) structure at room temperature and xylitol-bound (cyan) SruGI structure determined by serial millisecond crystallography. The two structures are almost identical with an r.m.s. deviation value of 0.112 Å. (d) B-factor representation of native and xylitol-bound SruGI determined by serial millisecond crystallography.

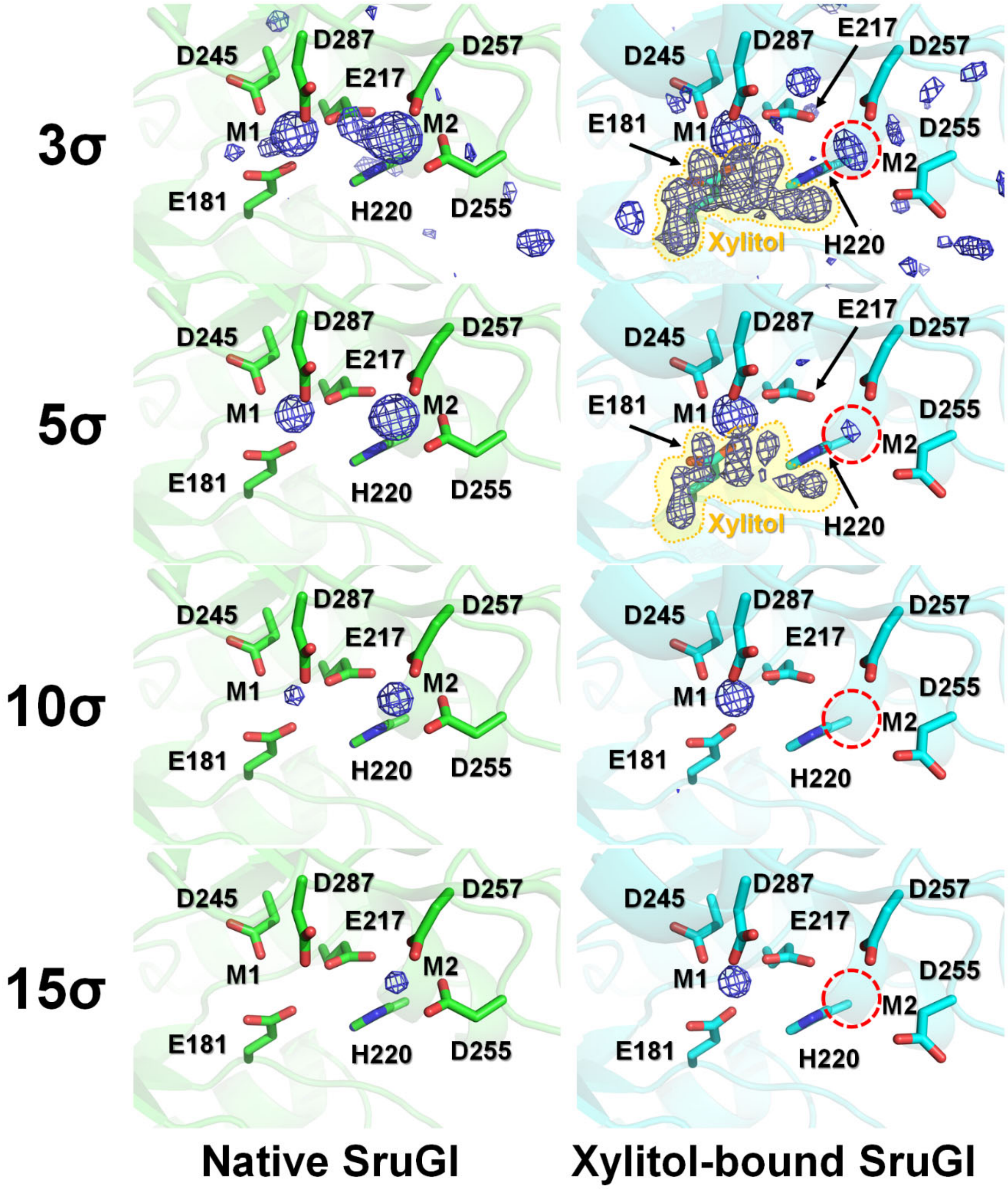

Supplementary Figure S4. The Fo-Fc omit electron density map (blue mesh) for the metal-binding site of (a) native and (b) xylitol-bound SruGI.

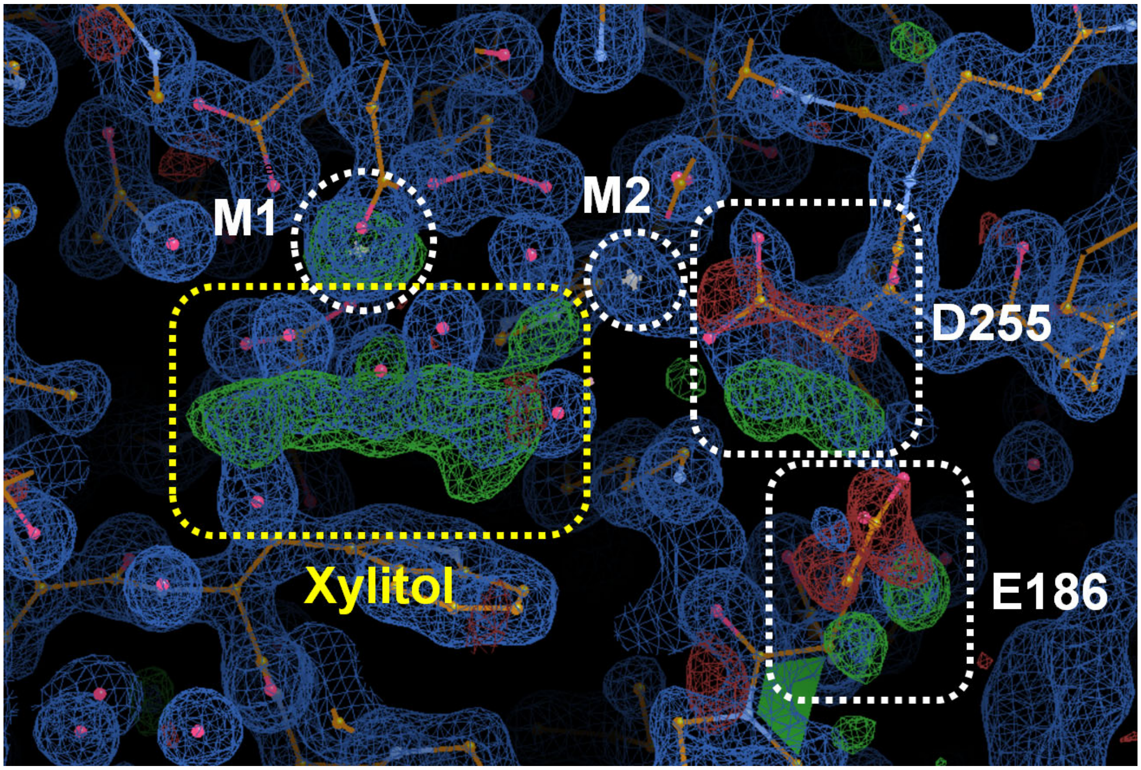

Supplementary Figure S5. 2Fo-Fc (blue mesh, 1σ) and Fo-Fc (green mesh, +3σ; red mesh, -3σ;) electron density map of xylitol-bound SruGI during the structure refinement using native SruGI as an initial model.

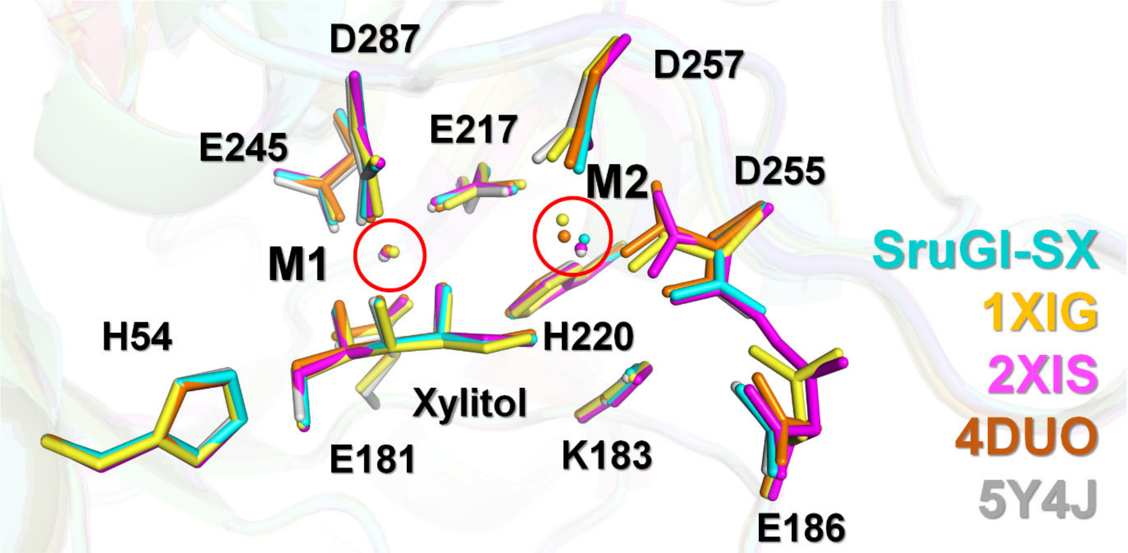

Supplementary Figure S6. Superimposition of xylitol-bound SruGI (SruGI-SX: cyan) by serial crystallography with crystal structure of xylitol-bound SruGIs (PDB code 1XIG: yellow, 2XIS: magenta, 4DUO: orange, and 5Y4J: gray) by traditional X-ray crystallography.

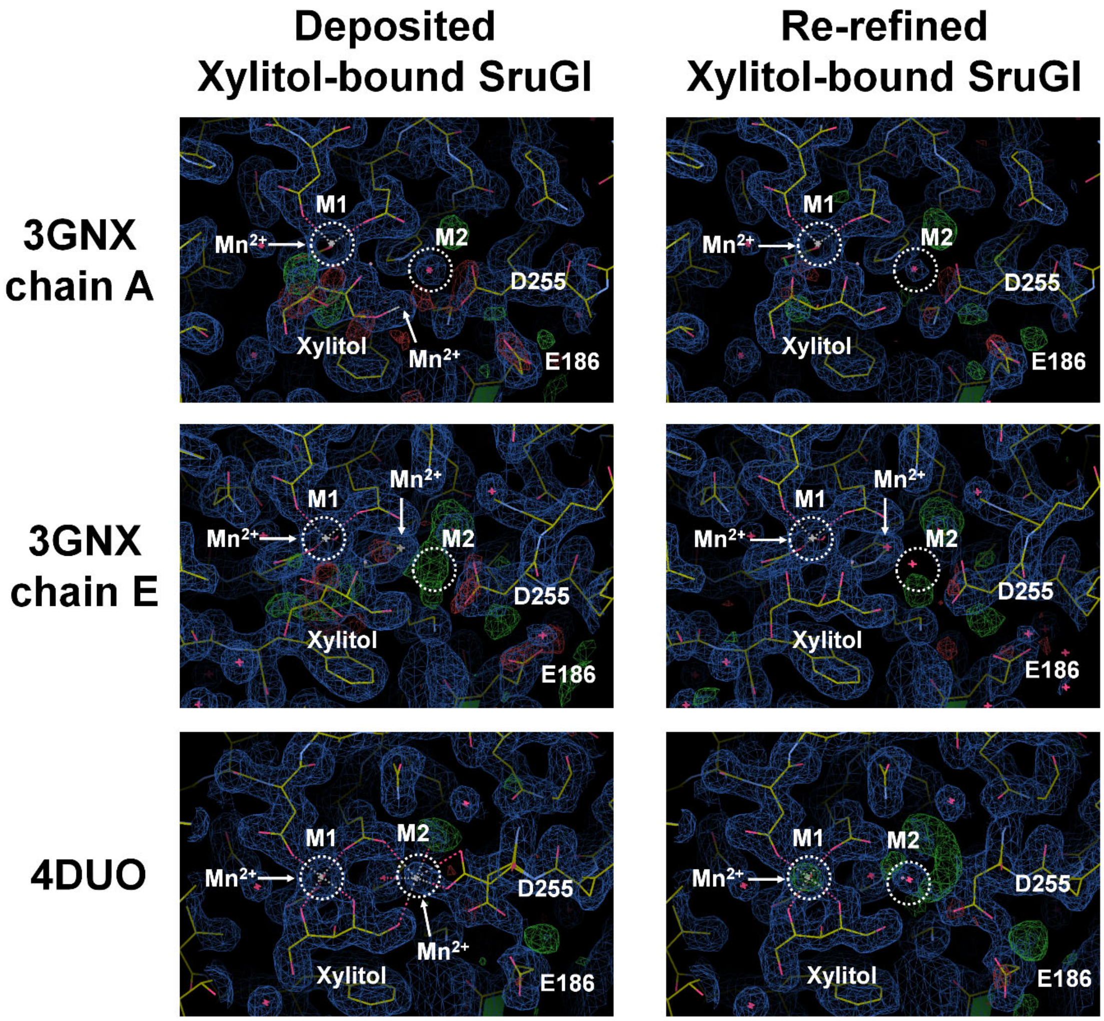

Supplementary Figure S7. Re-refinement of the deposited xylitol-bound SruGI. 2Fo-Fc (blue mesh, 1σ) and Fo-Fc (green mesh, +3σ; red mesh, -3σ;) electron density map of xylitol-bound SruGI (PDB code 3GNX and 4DUO).

Table S1. Atomic displacement parameter (ADP) of the re-refined metal-binding site of xylitol-bound SruGIs.

PDB Code	Modeled metal	B-factor (Å^2)			Geometry [2] [gRMSD(°)] [3]	
		Protein	M1 site (env) [1]	M2 site (env) [1]	M1 site	M2 site
SriGI-SX	Mg^{2+}	20.58	3.8 (12.8)	30 (18.6)	Octahedral (7)	Free
	Mn^{2+}		30.0 (12.8)	30.0 (18.7)	Octahedral (7)	Poorly coordinated
Re-refined 3GNX (Chain A)	Mg^{2+}	14.26	0.5 (10.9)	67.7 (18.3)	Octahedral (8.8)	Free
	Mn^{2+}		13.2 (10.5)	91.3 (15.6)	Octahedral (8.8)	Square Planar (4.8)
Re-refined 3GNX (Chain B)	Mg^{2+}	14.51	0.5 (11.2)	40.3 (25.9)	Octahedral (8.3)	Poorly coordinated
	Mn^{2+}		12.5 (10.8)	87.4 (22.5)	Octahedral (8.4)	Poorly coordinated
Re-refined 4DUO	Mg^{2+}	17.92	5.5 (13.5)	42.8 (17.9)	Octahedral (6.9)	Square planar (12.2)
	Mn^{2+}		21.9 (9.7)	73.1 (17.2)	Octahedral (7)	Square planar (25)
Re-refined 5Y4J	Mg^{2+}	12.17	6.6 (7.5)	17.5 (9.7)	Octahedral (6.1)	Free
	Mn^{2+}		16.6 (7.1)	31.9 (9.8)	Octahedral (5.9)	Square planar (5.6)

[1] Valence-weighted environmental average B factor in parentheses.

[2] Arrangement of ligands around the ion, as defined by the NEIGHBORHOOD algorithm.

[3] R.M.S. Deviation of observed geometry angles (L-M-L angles) compared to the ideal geometry.
